# Ethics Education for Successful Infectious Disease Control of COVID-19

**DOI:** 10.1007/s41649-020-00124-4

**Published:** 2020-05-22

**Authors:** Hannah YeeFen Lim

**Affiliations:** Division of Business Law, Nanyang Technological University, Singapore

**Keywords:** COVID-19, Social philosophy, Public health, Law, Standard of care

## Abstract

The infection rates of COVID-19 have been exponential in some countries despite the imposition of infectious disease control measures such as lockdowns and physical distancing, which form one of the basic principles of public health and infectious disease control. There have been significant problems with leaders and citizenry deliberately ignoring and not complying with such measures and which have directly resulted in sudden rises in infection numbers. Here, I show the nature and extent of the widespread problem and argue that the problem is in large part due to our modern society characterised by liberal individualism. I apply the philosophy proposed by philosopher Alasdair MacIntrye to show that one key underlying cause of the non-compliant behaviour of citizenry is due to modern liberal individualism that has deprived the modern nation state of the opportunities and authority for it to teach or to dictate what is the common good of the society as a whole to individuals in its community. This is the first time MacIntyre’s philosophy has been applied to public health, and this paper demonstrates the need for ethics education to counter-balance liberal individualism in order to contain and to prevent another pandemic and public health crisis in modern society.

Much critique and analysis has appeared on the possible missteps, missed opportunities and mismanagement of the current pandemic by authorities around the world. The purpose of this research is not to delve into those issues; rather, the question examined here is why, from a social philosophy perspective, so many individuals around the world were so unwilling to follow the directives on infection control measures such as to physically distance themselves from others and to stay at home—one of the basic principles of public health and infectious disease control; and also on the flip side, why were authorities in many jurisdictions, represented by individuals who make those decisions, so slow in implementing such measures, including isolation, quarantine and lockdowns, until arguably it was too late for that jurisdiction.

The practice of quarantine dates back to the fourteenth century to protect coastal cities from plague epidemics from visiting ships (CDC 2012). As the centuries rolled by, many jurisdictions continued the practice of quarantine. In the USA, protection against imported diseases initially fell under local and state jurisdiction with a variety of quarantine regulations implemented for arriving ships (CDC 2012). In 1878, the US Congress enacted federal quarantine legislation to contain outbreaks of yellow fever. This initial federal intervention was further strengthened in 1892 following outbreaks of cholera from ships arriving from Europe (CDC 2012). By 1921, the Federal government in the US had full control of the quarantine system (CDC 2012).

Similarly, in Australia, Sydney, being the gateway to Australia in the early colonial days, had a functioning “Quarantine Station” (QS) for more than 150 years. The QS was located at North Head, the entrance of Sydney Harbour, and from 1828, its main function was to quarantine new arrivals and prevent the introduction of infectious diseases into the country, although it was also used in 1881 to quarantine locals with smallpox, and similarly, also during the 1918–19 influenza pandemic (NSW Government Office of the Environment and Heritage, n.d.). In the UK, in 1666, the entire village of Eyam voluntarily isolated itself even though the villagers believed they faced near-certain death from the bubonic plague if they remained, but could cause the death of thousands in the big cities of Sheffield and Manchester if they left. The self-sacrifice lasted 14 months and was successful in containing the plague within the village (Purser Brown 2020).

Quarantine measures have been proven effective in the past, and even today, they remain an important and effective public health measure in infectious disease control.

## The Observable Global Problem

The current severe acute respiratory syndrome coronavirus 2 (SARS-CoV-2) originating in Wuhan, China, which causes COVID-19 disease and the current global pandemic was reported on 31 December 2019 to the World Health Organization (WHO) Country Office. It reached international headlines during January 2020 with countries such as Singapore immediately implementing measures to prepare for COVID-19 during January 2020. Many countries did nothing to prepare at that time.

As the health crisis developed, it was clear that there was no known treatment or vaccine, and it appeared to be highly contagious with thousands in China infected in the course of weeks causing deaths. Yet, governments and health authorities in many parts of the world were slow to take heed of the need to take action.

On 31 January 2020, Italy confirmed COVID-19 in two Chinese tourists, and the government declared a six-months state of emergency (New Zealand Herald 2020). The first community transmission seemed to have been confirmed on 20 February 2020, and the patient had led an active life even when he had symptoms and was obviously contagious (Horowitz et al. 2020). This should have sounded alarm bells for the Italian authorities; instead, on 27 February 2020, the leader of the governing Democratic Party Nicola Zingaretti urged people “not to change our habits” and posted a picture of himself clinking glasses for “an aperitivo in Milan” (Horowitz et al. 2020). Less than 10 days later in early March 2020, Italy had 5883 confirmed cases and 233 deaths (Horowitz et al. 2020), and it was reported that on 21 March 2020, there were 793 deaths, a one-day record that saw the country’s death toll increase to 4825 with the total number of confirmed cases of COVID-19 in Italy standing at 53,578 on 21 March 2020 (Channel News Asia 2020). On 27 March 2020, 1 month after Zingaretti’s online post, the number of confirmed cases in Italy stood at 86,498 with 9134 deaths (Washington Post 2020).

With the hospitals overflowing in the initially affected regions of Italy, the authorities took a piecemeal and at times contradictory and confusing approach (Horowitz et al. 2020) in attempts to contain the spread of COVID-19, by first implementing lockdowns in the northern Lombardy and Veneto regions on 22 February, followed by a closure of schools and universities on 4 March, and with nearly 5900 cases confirmed, on 8 March, Italy ordered a lockdown for 16 million people in the north whilst also closing museums and theatres nationally. By 10 March, there were nearly 7400 cases, and the lockdown was extended to the whole of Italy with limits on travel abroad and across regions. On 11 March, with nearly 12,500 confirmed cases, the government in Italy suspended nearly all commercial activity other than supermarkets and pharmacies (New Zealand Herald 2020).

Even as the lockdowns were implemented in Italy, many residents of Italy also flouted the rules; for example, people continued to exercise outdoors, much to the frustrations of the authorities, healthcare workers and the average citizen (New Zealand Herald 2020). Even more perplexing is the fact that tens of thousands were cited by the Italian police for breaking the lockdown rules (New Zealand Herald 2020).

A similar scene played out in Spain. On 8 March 2020, Spain’s Prime Minister Pedro Sanchez encouraged Spaniards to join a mass demonstration in support of International Women’s Day despite the lockdown that had already been imposed in northern Italy. Spain had 589 confirmed cases at that point. Around 120,000 people gathered for the event in Madrid, and by the next day, the number of confirmed cases had doubled, very likely due to the mass gathering. On 15 March, Sanchez ordered a national lockdown, but it may have been too late—in the 10 days after, Spain’s number of confirmed cases rose from 9000 to 50,000 (Sills and Lombrana 2020).

In Australia, another country that has been slow to implement effective infectious disease control measures such as lockdown, its beaches in Sydney were crowded with thousands of people on 21 March 2020, even when social distancing measures and restrictions on public gatherings were already in place. One beachgoer told the reporter “You can’t stop people from living. That’s the way I look at it. If it’s a nice day and I want to have a run and a swim, well I’m gonna go do it” with other beachgoers expressing the same sentiments (Taylor 2020). After the lifesavers on patrol at the beaches were harassed by beachgoers, the police moved to close all the beaches to implement the restrictions on mass public gatherings. Even after the beaches were closed, members of the public were still defiant and continued to head towards the beaches, making their way past barricades that had been set up (Pengilley and Thomas 2020). At that point in time, the state of New South Wales where Sydney is situated already had the highest number of cases in Australia with over 500 confirmed cases that had increased exponentially over the previous days and week (Pengilley and Thomas 2020). Finally, after the events at the beaches, on 22 March 2020, the state government announced that the state would go into lockdown by 24 March 2020 except for essential services, which strangely included not just supermarkets and petrol stations, but also hairdressers and beauticians, liquor stores and schools (Drevikovsky et al. 2020). However, 1 week later on 27 March, the number of confirmed cases in New South Wales had tripled to 1617, with many cases directly attributable to the beachgoers based on geographical location and movement tracing. Desperate to contain the spiralling number of infections, the Prime Minister Scott Morrison announced on 27 March that all returning travellers from overseas would be isolated for 14 days in hotels at the expense of taxpayers (Gladstone and Chung 2020). Even so, a group of 33 medical professionals out of a group of 77, returning from Chile, disobeyed police orders to be quarantined at an airport hotel and instead headed to the domestic airport terminal to fly home (Fitzsimmons 2020).

Elsewhere in the world, the story is similar. In the US, party-goers in New Orleans and Nashville were highly criticised for not abiding by the restrictions (Aguilera 2020), as were partying college students in Florida (BBC News 2020b). Even more alarming, from the middle of April 2020 onwards, many demonstrators in some US states blatantly ignored the lockdowns and the safe distancing measures in place and came out in force in the streets, some carrying guns and automatic weapons making their way into a Capitol building and attempted to enter the floor of the in-session legislative chamber (Beckett 2020), to protest the lockdowns and stay home orders (Dartunorro 2020). These protesters in over a dozen states (BBC News 2020a) across the US such as in Maryland, New Hampshire, Texas, Indiana, Nevada, Wisconsin, Ohio and California relied on claims of their supposed Constitutional rights not to be in lockdown (Straits Times 2020), whilst in some cities such as Denver, a few healthcare workers braved the protestors to stage their counter-protest by blocking the anti-lockdown demonstrations (Hutchinson 2020). Even as these protests were taking place on 21 April 2020, the US had 761,000 confirmed cases and more than 40,000 deaths (BBC News 2020a). As at 3 May 2020, the US had the highest number of confirmed cases in the world at 1,122,248 cases and the highest number of deaths in the world at 65,735 (CDC 2020).

On the extreme end, Singapore even saw several cases of Indonesians who already had symptoms of COVID-19 in Indonesia and had been hospitalised in Indonesia for pneumonia but who still nevertheless took flights to Singapore to utilise the at-the-time free for all treatment in Singapore for COVID-19, no doubt endangering the lives of those at the two airports and on the plane during their journeys (Aravindan and Geddie 2020). One such case arrived in Singapore in critical condition and had already been treated in an Indonesian hospital for pneumonia. He was immediately sent to a hospital from the airport in Singapore, but he subsequently became one of the first casualties from COVID-19 in Singapore (Liu 2020).

## The Cause of the Behaviour from the Perspective of Social Philosophy

The question must be asked why the leaders, authorities and individuals have behaved the way they have, despite clear evidence from overseas and pleas from healthcare workers and citizenry? Whilst many possible answers could be proffered such as the lack of preparedness on the part of authorities (see for example, Leslie et al. 2020), this cannot be said of the individuals. Indeed, even the authorities have the experiences of other countries as warning signs but many took no heed until it was arguably too late.

Was it just simply selfishness? Even selfishness needs a source, and that source, as I shall argue below is the individualism of modernity. In a sense, it is an unspoken and innately felt entitlement for individualism that characterises modernity and the modern society—an individualism and freedom often without corresponding responsibilities or duties, especially to others and society, sometimes even to oneself.

Philosopher Alasdair MacIntyre has warned against such a state of affairs in his works spanning several decades starting with *After Virtue*, MacIntyre (2007, 195) wrote:

For liberal individualism a community is simply an arena in which individuals each pursue their own self-chosen conception of the good life, and political institutions exist to provide that degree of order which makes such self-determined activity possible. Government and law are, or ought to be, neutral between rival conceptions of the good life for man, and hence, although it is the task of government to promote law-abidingness, it is on the liberal view no part of the legitimate function of government to inculcate any one moral outlook.

MacIntyre made the case that on the one hand that political society is envisaged as a series of communities such as family, workplace and neighbourhood and within these communities, human goods that are only available to us through common life and action are pursued. It is also through these communities that we learn, or at least can learn, that something is not good for me when it is not also a good for the community. We, as individuals, develop and discover ourselves in the context of continuously shaping and re-shaping our interactions with communities. From this, the goals of morality are seen to be positive even though negative rules are needed to set limits on what is an intolerable behaviour in the community life, that is, the common life. In this sense, political and moral life is envisaged predominantly in terms of the positive pursuit of goods for humans (MacIntyre 1980, 31).

On the other hand, however, especially in the modern age, many of us have grown up with an almost habitual thinking that human society is an arena where individuals and groups have rival and competing desires and goals, and these need to be protected from each other in order for each to pursue its own private aims and self-satisfactions. MacIntyre concludes that “We have no systematic way of reconciling these competing standpoints” (MacIntyre 1980, 31–32).

Given the current observable problems relating to COVID-19, MacIntyre looks to have been proven right as exemplified by the response to public health measures from both sides—the authorities and the citizenry. This is where the two competing standpoints come to a head, clashing precariously as thousands of lives are lost each day as countries attempt to flatten the infection curve and prevent the health systems from being overwhelmed, as well as to avoid putting healthcare workers in the impossible position of having to make impossible choices that have thus far resulted in none other than utilitarian and less than ethical decisions being made about choosing which patients should live or die, based on, for example, the age of a patient (Frakt 2020). This is notwithstanding that medical professionals have never been endowed with the legal power to decide which patient should live and which should die—indeed, the question for medical professionals should always be what is the best treatment for any particular patient. It is true that scarce resources had placed the healthcare professionals in these very difficult dilemmas, but they do have an ethical and professional responsibility to allocate scarce resources in a fair and equitable manner. In any event, it could also be argued that healthcare professionals may have been able to avoid making such difficult decisions if citizenry had complied with infectious disease control measures and the hospital systems had not been flooded with very ill patients during a very short period of time.

Following the philosophical thought of Aristotle and Thomas Aquinas, MacIntyre firmly believes in the importance of the human agent developing the intellectual and moral virtues and thereby becoming fully rational and free to choose morally what is good and best. For MacIntyre, the question of freedom becomes a question of education, in particular ethics and moral education, and human development and ultimately, a question of political systems too.

MacIntyre, writing in and about the US for most of his career, holds that the key way for one to become a mature and wise adult with independent good practical reasoning who can consistently discern what is good and best to do is through the moral and ethics formation and education provided by communities that seek the good together as a community for the further good of the community (MacIntyre 2007, 220; MacIntyre 1998). The deprivation of opportunities for such communal education about the good would appear to be, in MacIntyre’s view, prevalent in liberalism (MacIntyre 1998). This is the reason why MacIntyre rejects liberalism as an ideology because he argues that the rise of liberalism has resulted in the fabric of morality in society being torn apart—presumably he was writing about the US where he was based (MacIntyre 1980, 33). Liberal individualism asserts the freedom of autonomous adults to pursue their own individual ends, and protects the individual from community-imposed determinations of the good with the end result that the government becomes the protector of the individual autonomy, at the sacrifice of the true common good (MacIntyre 2007). This is exactly what we have witnessed in 2020 with governments in many countries, including the US, struggling to assert their authority over citizenry to isolate, and to lockdown for the common good of the entire community, which, of course, also includes the healthcare system.

MacIntyre advocates a politics of the true common good that liberal individualism cannot match. In the modern liberal nation state, MacIntyre posits that the political order is merely instrumental to secure a social order within which individuals may pursue their own particular ends with the result that the common good is just simply a summation of individual goods, a superficial conception of the common good that is both minimalist and individualist and which he rejects (MacIntyre 1998). This kind of summation of individual goods could be reflected in notions of utilitarian common good, where not only the interests of the elderly patient may yield to that of a younger patient deemed to be more healthy, and more likely to survive and hence more deserving of the use of a ventilator (Frakt 2020) but also where a healthy patient may be sacrificed to save many other patients.

This minimalist and individualist notion of the common good is also exactly what the world has seen in the modern day attempts at infectious disease control to protect public health in many jurisdictions around the world, with leaders and individuals blatantly defiant in refusing to act for the common good in the Aristotelian and MacIntyrean senses. This must clearly be seen as one of the failures of the liberal individualism of the modern state in that it cripples one key pillar of the basic principles of public health and infectious disease control—the pillar of physical distancing, lockdowns and even quarantine.

## Ethics Education

Taking up MacIntyre’s precept, ethics formation and education should be provided by communities that seek the good together as a community for the further good of the community. This is still fairly broad and open-ended and would mean that what each community would seek together as the good may differ from community to community. However, is it possible to find some commonality that is teachable? And at what stage in one’s life should ethics education begin?

It may well be that some will argue that the earlier children are taught ethics, the more effective it will be. This line of thinking is somewhat akin to parents teaching their young children to say “thank you” and “please” at an early age in order to inculcate good manners and good habits. It may be the case however that teenagers, having attained some maturity and practical reasoning abilities, are more suitable to ethics education. Certainly by the time one enters university, one would benefit from ethics education and some universities do mandate undergraduates take general education courses such as ethics. However, if ethics education is left until university, those who do not attend university would be left out. Whilst civic bodies could step into the gap to educate working adults, there may be challenges, such as time and other constraints. In the final analysis, ethics education may be best begun in schools and continued at university level.

Returning to the Aristotelian-MacIntyrean common good, one common thread in the ethics education of communities might be to emphasise that the individual exists within and not apart from society, and thus, notions of absolute freedom are untenable to the concept of the common good. Communities would do well to uphold the dignity, unity and equality of all people, especially those most vulnerable; and utilitarian thoughts that may result in one individual being sacrificed for another should be avoided. The common good should be taught as an ideal that is indivisible because it is only attainable together as a community and can only be increased together as a community. The common good requires a constant effort to seek the good of others as though it were one’s own good.

Some may argue that the common good will quash the individual’s ability to achieve fulfilment or happiness. On the contrary, Aristotle posited that *eudaimonia* or happiness or fulfilment is only possible if one’s actions are virtuous in accordance with the Golden Mean, a mean that lies in between two extremes, or vices, one of excess and one of deficiency. In particular, the virtue of justice, which pertains to others and not just oneself, is an absolute mean with the result that if one were to act unjustly, to benefit oneself for example, then one would not be achieving fulfilment or happiness (Aristotle 340BC, 1908).

Admittedly, ethics education may not be sufficient to convince all individuals to act for the common good, and it may be that in order for the common good to be upheld, the state may need to be given legal power to act in the public interest to mandate the common good in order to deal with incalcitrant individuals who still hold to individualism.

For some communities, in deriving the content of the ethics education, the COVID-19 pandemic may be an opportunity to re-evaluate the social dynamics. Could it be, for example, that the individualistic attitude is also driven by consumerist culture and perhaps to examine its foundations? Some may question whether the over-possession of material goods, which is directed at the individual, is excessive.

## Conclusions

In sum, the catastrophically high number of deaths from COVID-19 as seen around the world in February and March 2020 is to a large extent systemic and indicative of the liberal individualistic modernity that society has arrived at. If the village of Eyam could control the bubonic plague in 1666, one must seriously consider the need for ethics education now and in the future to educate on, not only the need for the Aristotelian-MacIntyrean common good to be discerned and observed but also to counter-balance liberal individualism in order to prevent another public health crisis in modern society.
